# Analysis of risk factors related to the progression rate of hemifacial spasm

**DOI:** 10.3389/fneur.2024.1357280

**Published:** 2024-03-28

**Authors:** Fei Xu, Pengju Gu, Huan Yuan, Li Jiang, Yanfeng Xie, Quanhong Shi, Yan Zhan

**Affiliations:** Department of Neurosurgery, The First Affiliated Hospital of Chongqing Medical University, Chongqing, China

**Keywords:** hemifacial spasm, progression rate, facial nerve angel, root entry zone, APOE ε4, magnetic resonance tomography angiography

## Abstract

**Introduction:**

Although there have been many researches on the etiology and risk factors with the onset of hemifacial spasm, researches on the risk factors related to progression rate are limited. This study aims to analyze the risk factors related to the progression rate of hemifacial spasm.

**Methods:**

The study enrolled 142 patients who underwent microvascular decompression for hemifacial spasm. Based on the duration and severity of symptoms, patients were classified into rapid progression group and slow progression group. To analyze risk factors, univariate and multivariate logistic regression analyses were conducted. Of 142 patients with hemifacial spasm, 90(63.3%) were classified as rapid progression group, 52(36.7%) were classified as slow progression group.

**Results:**

In the univariate analysis, there were significant statistical differences between the two groups in terms of age of onset (*P* = 0.021), facial nerve angle (*P* < 0.01), hypertension (*P* = 0.01), presence of APOE ε4 expression (*P* < 0.01) and different degrees of brainstem compression in the Root Entry Zone (*P* < 0.01). In the multivariable analyses, there were significant statistical differences between the two groups in terms of age of symptom onset (*P* < 0.01 OR = 6.591), APOE ε4 (*P* < 0.01 OR = 5.691), brainstem compression (*P* = 0.006 OR = 5.620), and facial nerve angle (*P* < 0.01 OR = 5.758). Furthermore, we found no significant correlation between the severity of facial spasms and the progression rate of the disease (*t* = 2.47, *P* = 0.12>0.05).

**Conclusion:**

According to our study, patients with facial nerve angle ≤ 96.5°, severer compression of the brainstem by offending vessels, an onset age > 45 years and positive expression of APOE ε4, may experience faster progression of hemifacial spasm.

## Introduction

Hemifacial spasm (HFS) is a neuro-muscular disorder characterized by intermittent and involuntary contractions of facial muscles on one side of the face. The contractions typically initiate from the orbicularis oculi muscle and gradually spread to other facial expression muscles with a minority of cases originating from the orbicularis oris muscle (1). However, the disease progression varies individually, with some patients experiencing rapid extension of symptoms involving a major portion of the affected side, while others exhibiting slow extension of symptoms over an extended period. The debilitating nature of HFS causes significant psychological distress (2, 3) and leads to affect the quality of life for the affected individuals (4). Currently, it has been reported that progression rate of hemifacial spasm is related to the postoperative outcome of patients. Lee et al. (5) reported rapid progression led to worse clinical outcomes, such as more cases with persistent spasm postoperatively.

In this study, we aim to analyze the risk factors related to the progression rate of hemifacial spasm, providing theoretical support to guide clinicians and patients on microvascular decompression (MVD) surgery. MVD surgery is widely accepted to carry out to cure HFS. Concerning about the possible risk of MVD surgery, and worse clinical outcomes in rapid progression rate patients, prediction of spasmic progression rate is a great need to determine whether and when to perform MVD surgery.

## Materials and methods

### General information

This retrospective study collected data by reviewing medical records of 144 consecutive patients who underwent microvascular decompression (MVD) for HFS performed by a single surgeon (Y.Z.) between November 2019 and May 2021. Two of 144 were excluded due to lack of imaging data. This study was approved by the ethical committees of the First Affiliated Hospital of Chongqing Medical University. Informed consent was obtained from all the individual participants included in the study. Our research collected detailed preoperative information, including gender, affected side, hypertension, diabetes, age of symptom onset, duration of spasms, severity grading of spasms, number of offending vessels, whether the offending vessels includes the vertebral artery. To record disease progression, we adopted a severity grading system based on Lee et al. which divided the severity of HFS into four grade: Grade I - limited to periorbital region on one side; Grade II - involving other muscle groups on the same side of the face, such as the orbicularis oris muscle or zygomaticus muscle; Grade III - affecting vision due to frequent spasms; Grade IV - affecting vision and causing bilateral facial asymmetry (6, 7). However, there is currently no unified standard for calculating the progression rate of HFS patients. The average time taken to progress one grade among 142 patients was 1.61 years/grade in our research. Therefore, we defined rapid progression group as those with a progression rate of <1.61 years/grade, and slow progression group as those with a progression rate more than 1.61 years/grade.

### Inclusion and exclusion criteria

Patients who met the clinical presentation of HFS, had a duration longer than 3 months, excluded from secondary lesions by CT or MRI scans, and showed offending vessels on preoperative magnetic resonance tomography angiography (MRTA) were included.

Patients who had a duration shorter than 3 months, a previous diagnosis of psychiatric disorders, stroke, encephalitis, dementia, multiple sclerosis, or secondary hemifacial spasm were excluded.

### Characteristic

#### Facial nerve angle

**Figure 1:**
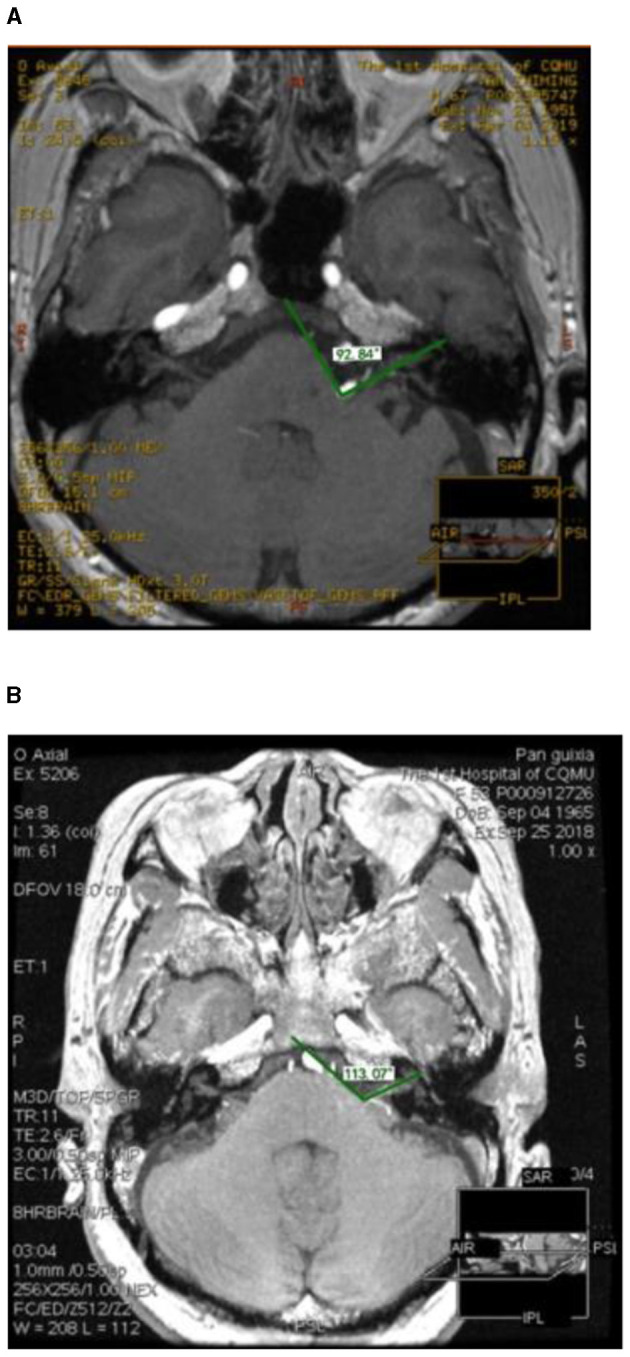
The green lines show the angles between the inner margin of the facial acoustic nerve and the anterior surface of the brainstem at the point of nerve exit. Facial Nerve Angel ≤ 96.5° **(A)**. Facial Nerve Angel >96.5° **(B)**.

The facial nerve angle was defined as the angle between the inner margin of the facial acoustic nerve and the anterior surface of the brainstem at the point of nerve exit (8). Shein et al. found that patients with smaller facial nerve angles had a higher incidence of hemifacial spasm (8). Based on this, we speculated whether there was a critical value for the facial nerve angle that correlated with the progression rate of HFS patients. Therefore, we measured the average facial nerve angle of the 142 patients. The average angel of 142 patients was 96.50°. We divided them into two categories: ≤ 96.5° and >96.5° (Figures 1A, B).

#### Brainstem compression in the REZ

Root Entry Zone (REZ) is the area where the cranial nerve comes out of the brainstem. It is widely accepted that offending vessels compress the facial nerve REZ area (9). However, it is important to note that not every part of the REZ area under compression results in hemifacial spasm symptoms. Within the REZ area, there are specific anatomical regions referred to as “sensitive sites,” which are situated 1–3 mm beyond the exit point of the facial nerve root (10). We hypothesized that the severity of compression on these sensitive sites might influence the progression rate of hemifacial spasm in patients. Given that these regions are intimately connected to the brainstem within the REZ area, we employed Magnetic Resonance Tomography Angiography (MRTA) to assess the degree of brainstem compression in the REZ area, particularly focusing on the facial nerve root's sensitive sites. We categorized this compression as follows.

**Figure 2:**
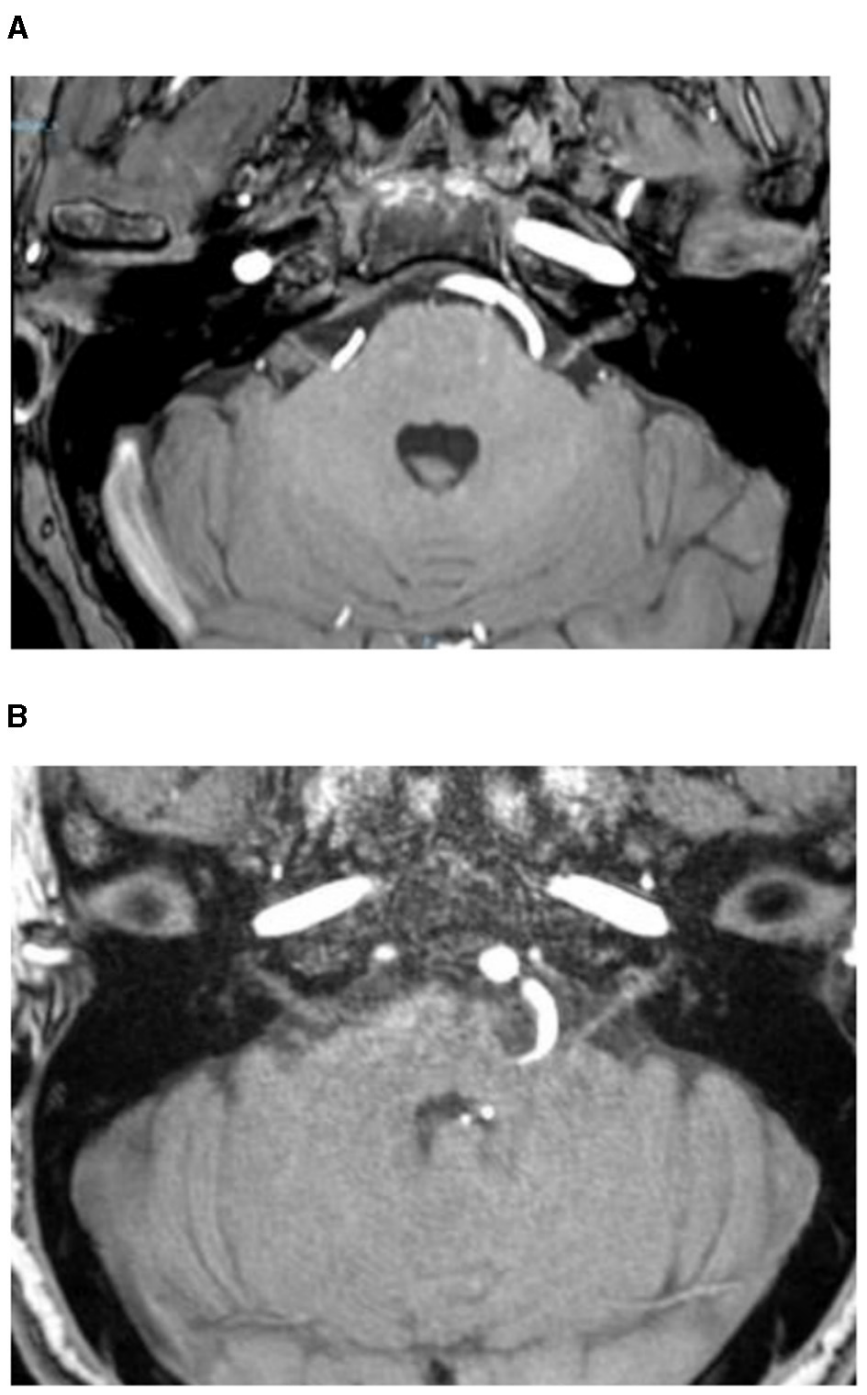
Offending vessels in direct contact with the REZ area of the brainstem, resulting in compression without brainstem deviation (Grade I) **(A)**, with brainstem deviation (Grade II) **(B)**.

Grade I: Offending vessels in direct contact with the REZ area of the brainstem, resulting in compression without brainstem deviation (Figure 2A).

Grade II: Offending vessels in direct contact with the REZ area of the brainstem, leading to compression of the brainstem and subsequent deviation (Figure 2B).

#### APOE ε4

Our previous research found a higher incidence of HFS in patients with the APOE ε4 genotype (11). Therefore, we hypothesized that APOE ε4 carriers may experience a faster progression of HFS. The APOE genotype of each patient was determined using quantitative fluorescence polymerase chain reaction (QF-PCR). Based on the presence of the APOE ε4 allele in the genotypes, patients were classified as either APOE ε4 carriers or non-carriers. Subsequently, the positive rates of APOE ε4 genotype carrier status were calculated separately for the rapid and slow progression groups to determine if there were any differences between the two groups.

#### Hypertension, diabetes, and age of symptom onset

A research team from Japan found a possible association between the severity of arterial sclerosis in elderly males and the onset of hemifacial spasm (12). Arterial sclerosis has been confirmed to be closely related to hypertension, diabetes, and age of symptom onset (13). Therefore, we speculated that hypertension, diabetes, and age of symptom onset might be associated with the progression rate of HFS. We wanted to know if there was a threshold age of onset that was associated with the rate of disease progression. In our research, the average age of onset among the 142 patients was 45 years. They were then divided into two categories: ≤ 45 years and >45 years.

#### The vertebral artery

Patients with offending vessels involving the vertebral artery have a higher disease severity, surgical difficulty, and postoperative risk of delayed facial palsy compared to those with other offending vessels (14). Therefore, we hypothesized that patients with offending vessels involving the vertebral artery might have a faster progression rate of the disease. To investigate this, we separately calculated the positive rates of the involvement of the vertebral artery as the offending vessel in the rapid and slow progression groups, to observe if there were any differences between the two groups.

#### The severity of hemifacial spasm

We observed variations in the severity of spasms among patients with different progression rates. Referring to the Samsung Medical Center Grading System [SMC grade (6, 7)] criteria for HFS, we classified the severity of spasms into mild (SMC Grades I-II) and severe (SMC Grades III-IV). We counted the number of patients in the rapid and slow progression groups who exhibited mild or severe symptoms to assess if there were any differences between the two groups.

### Statistical methods

All analyses were performed using SPSS 27.0 software. Continuous data were presented as mean ± standard deviation (x ± s) and were analyzed by using independent samples *t*-test. Categorical data were expressed as proportions (%) and were analyzed by using Pearson's chi-square test, with R × C contingency table method employed (Fisher's exact test used when the expected count was below 5 in more than 1/5 of the cells or when the expected count was <1). A correlation analysis was performed to identify the factors correlated with progression rate of facial spasm. A significance level of α = 0.05 was used for all tests. Drawing in this study was performed using GraphPad Prism8.

## Results

**Figure 3:**
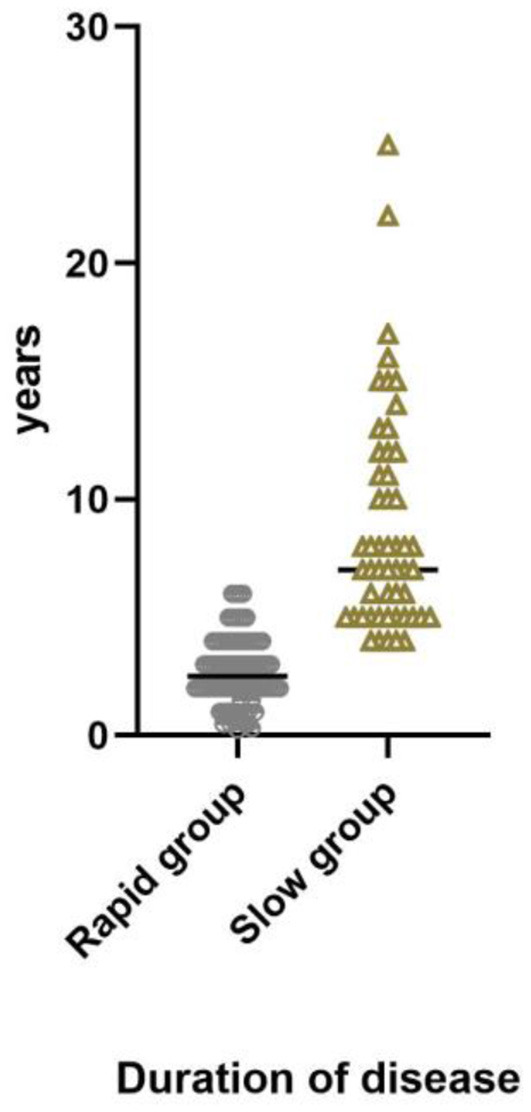
Duration of disease in rapid progression group, median 2.5 years, range 0.25–6 years. In slow progression group, median 7 years, range 4–25 years.

**Figure 4:**
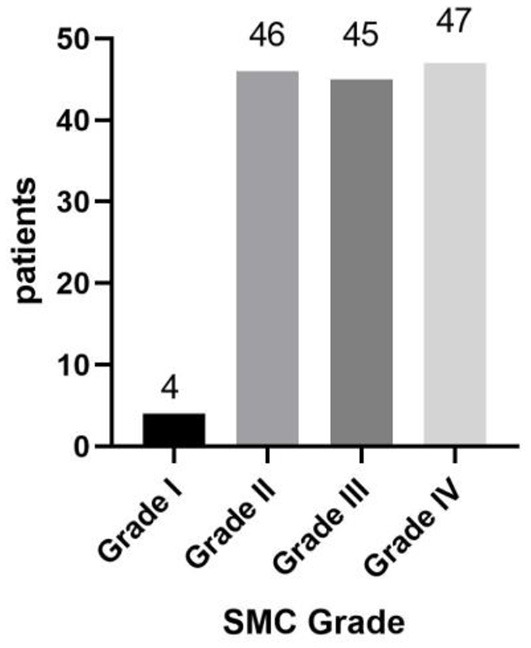
From mild to severe, the symptom was divided into four grades. Grade IV was the most severe. Grade I: 4 patients; Grade II: 46 patients; Grade III: 45 patients; Grade IV: 47 patients.

Of 142 patients, 90 (63.3%) were in rapid progression group and 52 (36.6%) were in slow progression group. The mean duration of disease in rapid progression group was 2.67 years (median 2.5 years, range: 0.25–6 years); in slow progression group, the mean duration was 8.78 years (median 7 years, range: 4–25 years) (Figure 3). Of 142 patients, 4 (2.8%) were in Grade I, 46 (32.4%) were in Grade II, 45 (31.7%) were in Grade III and 47 (33.1%) were in Grade IV (Figure 4).

### Differences between rapid progression group and slow progression group

Within rapid progression group, 36 patients (40.0%) were male, while in slow progression group, there were 20 male patients (38.5%). Similarly, hypertension was identified in 11 patients (12.2%) in rapid progression group and in 15 patients (28.8%) in slow progression group. The occurrence of diabetes was observed in 4 patients (4.4%) in rapid progression group and 4 patients (7.7%) in slow progression group. Among rapid progression group, 41 patients (45.5%) experienced affliction on the left side. In slow progression group, 32 patients (61.5%) were affected on the left side. Specifically, in rapid progression group, 29 patients (32.2%) had offending vessels involving the vertebral artery, while in slow progression group, 21 patients (40.4%) had offending vessels involving the vertebral artery. In terms of brainstem compression in the REZ area, rapid progression group had 35 patients (38.9%) presenting brainstem compression, while slow progression group had only 5 (9.7%). In rapid progression group, 55 patients (61.1%) experienced an onset age of >45 years. In slow progression group, 21 patients (40.4%) were >45 years old when they initiated facial spasm. Furthermore, in rapid progression group, 51 patients (56.7%) had a facial nerve angle ≤ 96.50 degrees. In slow progression group, 20 patients (38.5%) had a facial nerve angle ≤ 96.50 degrees. Rapid progression group had an average of 1.40 ± 0.54 offending vessels, while slow progression group exhibited an average of 1.35 ± 0.48 offending vessels. Notably, rapid progression group exhibited positive APOE ε4 expression in 59 patients (65.6%), whereas in slow progression group, positive expression was observed in 11 patients (21.2%) (Table 1).

**Table 1:** Comparison of clinical characteristics between rapid progression group and slow progression group.

**Characteristic**	**Rapid progression group (*n =* 90)**	**Slow progression group (*n =* 52)**	***x*^2^/F**	***P*-value**
Gender, female/male	54 (60.0%)/36 (40.0%)	32 (61.5%)/20 (38.5%)	0.33	0.86
Hypertension, yes/no	11 (12.2%)/79 (87.8%)	15 (28.8%)/37 (71.2%)	6.09	0.01^*^
Diabetes, yes/no	4 (4.4%)/ 86 (95.6%)	4 (7.7%)/48 (92.3%)	0.12	0.67
Affected side, left/right	41 (45.5%)/49 (54.5%)	32 (61.5%)/20 (38.5%)	5.39	0.06
Offending vessel include VA, yes/no	29 (32.2%)/61 (67.8%)	21 (40.4%)/31 (59.6%)	0.96	0.33
Brainstem Compression in the REZ area, Grade I/ Grade II	55 (61.1%)/35 (38.9%)	47 (90.3%)/5 (9.7%)	13.95	<0.01^*^
Age at symptom onset, years > 45 years/ ≤ 45 years	55 (61.1%)/35 (38.9%)	21 (40.4%)/31 (59.6%)	5.71	0.021^*^
Facial Nerve Angel, ≤ 96.50°/>96.50°	51 (56.7%)/39 (43.3%)	20 (38.5%)/32 (61.5%)	4.39	0.036^*^
Number of offending vessels	1.40 ± 0.54	1.35 ± 0.48	0.59	0.51
APOE ε4, positive/negative	59 (65.6%)/31 (34.4%)	11 (21.2%)/41 (78.8%)	25.99	<0.01^*^

VA, vertebral artery; REZ, Root Entry Zone. ^*^p < 0.05.

**Figure 5:**
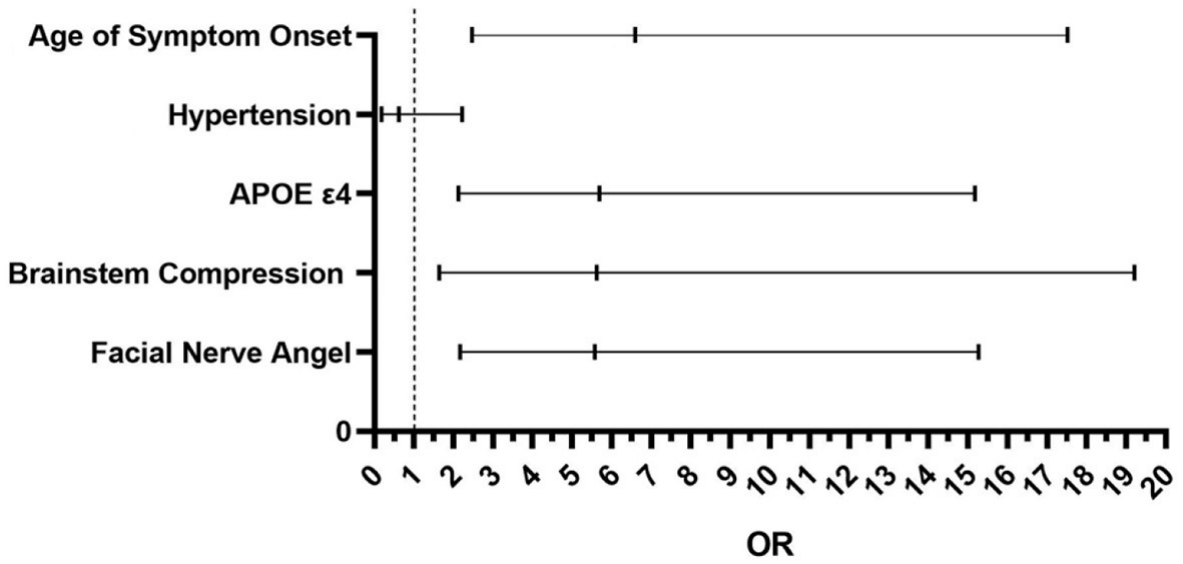
The result of multivariate logistic regression analysis between the two groups. Age of symptom onset *P* < 0.01 OR = 6.591; Hypertension *P* = 0.47 > 0.05. OR = 0.627; APOE ε4 *P* < 0.01 OR = 5.691; Brainstem Compression *P* = 0.006 <0.05 OR = 5.620; Facial Nerve Angle *P* < 0.01 OR = 5.758.

In the univariate analysis, there were significant statistical differences between the two groups in terms of age of onset (*P* = 0.021 <0.05), facial nerve angle size (*P* < 0.01), hypertension (*P* = 0.01 <0.05), presence of APOE ε4 expression (*P* < 0.01), and different degrees of brainstem compression in the REZ (*P* < 0.01). However, there were no significant statistical differences between the two groups in terms of gender, affected side, involvement of the vertebral artery, number of offending vessels, or diabetes. In multivariate logistic regression analysis result showed that patients with the age of symptom onset being >45 years was positively correlated with the progression rate (*P* < 0.01). Among patients with hemifacial spasm, those who expressed the APOE ε4 genotype had a fivefold higher progression rate compared to non-expressers (*P* < 0.01). Furthermore, more severe brainstem compression had a faster progression rate (*P* = 0.006 <0.05). When the facial nerve angle was ≤ 96.5°, the disease progressed faster (*P* < 0.01) (Table 2 and Figure 5).

**Table 2:** Multivariable analysis for factors associated with symptom progression rate.

**Characteristic**	**Odds ratio (95% confidence interval)**	***P*-value**
Age at symptom onset	6.591 (2.479–17.522)	*P <* 0.01^*^
Hypertension	0.627 (0.177–2.227)	*P =* 0.47
APOE ε4	5.691 (2.134–15.178)	*P <* 0.01^*^
Brainstem compression in the REZ area	5.620 (1.644–19.218)	*P =* 0.006^*^
Facial nerve angel	5.758 (2.171–15.270)	*P <* 0.01^*^

^*^P < 0.05.

### The severity of hemifacial spasm

**Figure 6:**
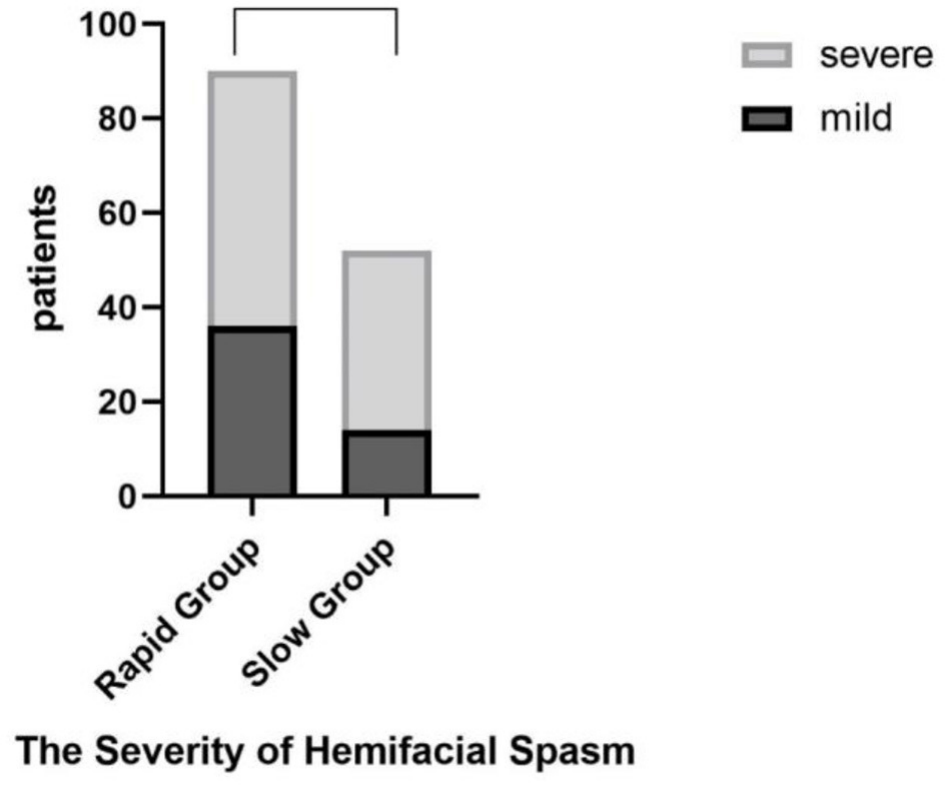
The severity of hemifacial spasm has no difference between the two groups.

Furthermore, we found no difference between the severity of facial spasms and the progression rate of the disease (*t* = 2.47, *P* = 0.12 > 0.05) (Figure 6).

## Discussion

In our research, we analyzed the possible factors influencing the progression rate of patients with hemifacial spasm by dividing them into rapid progression and slow progression groups. Our analysis revealed that facial nerve angel, hypertension, APOE ε4 genotype, and brainstem compression were shown to be associated with the rate of progression of hemifacial spasm in univariate analysis. Gender, affected side, number of offending vessels, involvement of the vertebral artery, diabetes and hypertension were not associated with the progression rate. A logistic regression showed that patients with more severe brainstem compression in the REZ (Grade II) exhibited a faster progression rate. Additionally, patients with age of symptom onset >45 years, facial nerve angel ≤ 96.5°and carrying the APOE ε4 genotype showed a faster rate of progression. Furthermore, we also found that there was no significant correlation between the progression rate of patients and the severity of patients' hemifacial spasm.

Previous study has shown that younger age at surgery, older age at symptom onset, and absence of intraoperative indentation on the facial nerve were associated with rapid progressive HFS (5). However, indentation on the facial nerve could only be discovered during surgery. Compared with previous study, the risk factors we analyzed could be obtained from patient's preoperative imaging findings, simple clinical history and lab test. Our findings might have practical significance. Especially for patients who are still in the early stage of facial spasm, predicting progression rate of disease is extremely significant to evaluate between the distress brought by the disease and the risk of surgery. According to the brainstem compression in the REZ in MRI image, APOE genotytpe and other factors that demonstrated in this study, progression rate of HFS could be predicted. Patients who are predicted to have slow progression rate might have sufficient time to follow up without significant distress. For patients with the above related factors, who might have rapid progression rate, it is recommended to porform MVD surgery as soon as possible to avoid further suffering.

### Age of symptom onset

Patients with an age of symptom onset >45 years had a faster progression rate. Conte et al. (15) showed that the spread latency correlated with disease duration and inversely correlated with age of onset. They explained that this result implied that age negatively impacts the course of the disease and that aged facial nerve fibers were more prone to develop spread. Jannetta et al. (16) had identified aging as a factor associated with a higher risk of elongated arteries, and brain aging, ultimately increasing the risk of neurovascular compression. Miki Ohta et al. (12) indicated that arterial changes associated with atherosclerosis in elderly patients may contribute to the development of hemifacial spasm. Based on the relevant researchers, we speculate that this may be due to two points: (1) aging facial nerves are more prone to demyelinating changes and faster spread. (2) age-related arterial stiffness leads to more severe atherosclerosis in blood vessels. Therefore, age plays a crucial role in the progression rate of hemifacial spasm.

### Facial nerve angle

In patients with a facial nerve angle ≤ 96.5°, the disease progressed faster. Smaller angle indicates a potentially more severe compression of the facial nerve root by offending vessel. Zhu et al. (17) observed that patients with hemifacial spasm had significantly smaller facial nerve angles compared to the unaffected side. And patients who experienced recurrence after microvascular decompression surgery had smaller facial nerve angles compared to the non-recurrence group. This can be explained by the fact that a smaller facial nerve angle increases the likelihood of vascular compression on the facial nerve in the REZ. According to the “demyelination theory” proposed as a possible mechanism for the development of hemifacial spasm (18–21), it is hypothesized that a smaller facial nerve angle leads to more severe compression of offending vessels on the facial nerve in the REZ area, that ultimately results in a faster progression of hemifacial spasm.

### APOE ε4

The APOE gene consists of 4 exons and 3 introns, totaling 3597 nucleotides. APOE has 3 alleles (APOE ε2, APOE ε3, and APOE ε4) and 6 phenotypes (ε2/ε2, ε2/ε3, ε2/ε4, ε3/ε3, ε3/ε4, and ε4/ε4) (22). Among them, APOE ε3 is the most common wild-type allele in the population. APOE ε2 and ε4 carry mutations and, with ε4 being considered a negative regulatory factor in many diseases (23, 24). Additionally, ε4 may promote the release of inflammatory factors (25) and play a role in the pathological processes of cerebrovascular and demyelinating diseases (26–28). Previous research has found that people carrying APOE ε4 have a higher incidence rate of HFS (11, 29). Research has found that APOE ε4 can accelerate the pathological process by interfering with neuronal and myelin regeneration (23, 24). Pathological changes such as demyelination, axonal loss, and Schwann cell proliferation can occur in HFS patients. We speculate that the repair of myelin in the compressed area may be disrupted in APOE ε4 gene carriers, making abnormal conduction of electrical signals more likely to occur. Moreover, APOE ε4 allele promotes the secretion of inflammatory factors and aggravates neuroinflammation. Thus, APOE ε4 allele interferes with the repair of myelin sheath in the compression area and results in electrical signals being more prone to ectopic conduction. It is reasonable to explain that patients with APOE ε4 positive expression have a faster rate of disease progression.

### Brainstem compression in the REZ

Through our data analysis, we found that the more severe the compression of the brainstem in the REZ area, the faster the progression rate of hemifacial spasm. Studies have found that primary hemifacial spasm is triggered by offending vessels, which lead to increased excitability of facial nerve in some focal areas through mechanical factors (indirect stretching) (30). The increased excitability can spread from one branch of nerve to another branch and result in faster excitation conduction (31). At the same time, it has been found that the facial nerve root may increase the expression of sodium ion channel Nav1.8 on the neuron membrane when it is pulled by the instrument, and the resting membrane potential also depends on sodium conductance, especially through the persistent sodium channel. Other sodium channels are also involved in the generation of action potentials (transient sodium channels). In the pressurized facial nerve in primary HFS rat model, one of the sodium ion channels, Nav1.8 channel, was found to be overexpressed, which affected the resting potential of neurons and increased the excitability of the facial nerve nucleus (30). We hypothesize that, mechanical compression by offending vessels lead to overexpression of sodium channels and result in faster conduction, and the more severe brainstem compression in the REZ region, the faster symptom progression will be.

### Other characteristic

In our data analysis, we found that gender and the affected side did not have a significant impact on the progression rate of hemifacial spasm in the two patient groups with differing rates of progression. Nurminen et al. (32) conducted a statistical analysis and reported a higher likelihood of hemifacial spasm in females, with a higher occurrence of left-sided hemifacial spasm. However, they did not provide a deeper explanation for his observations, and the relationship between hemifacial spasm and gender or affected side remains inconclusive. Some studies have suggested that postoperative complications were more common happened in cases of hemifacial spasm involving the vertebral artery in the offending vessels and those patients also exhibited a higher rate of delayed recovery after surgery (33, 34). However, there is no definitive evidence to suggest that the number of the offending vessels correlates with the severity of compression in the REZ area. Therefore, the presence of the vertebral artery in the offending vessels, or an increased number of the offending vessels, does not establish a clear relationship with the progression of hemifacial spasm. While there is established research indicating that a long history of diabetes may lead to atherosclerosis, a study by Ohta et al. (12) found no significant correlation between atherosclerosis and the occurrence of hemifacial spasm. Currently, there is also no evidence to suggest a relationship between diabetes and the incidence of hemifacial spasm.

### The severity of hemifacial spasm

We did not find a significant correlation between the rate of progression and the severity of hemifacial spasm (HFS) in our study. We initially thought that areas surrounding the facial nerve with faster progression may exhibit higher levels of pro-inflammatory cytokine release and slower repair of demyelination, leading to more severe symptoms in patients (29). Additionally, compression or stretching of the offending vessels on the facial nerve may not only stimulate excessive expression of the Nav1.8 channel but also cause hyperexcitability and “cross-excitation” between axons, resulting in more severe symptoms of facial spasm (30). However, our study did not find any differences between the two groups, which may be related to the limited number of cases collected.

### Limitations

Our study has certain limitations. Firstly, this study is a single-center retrospective and small sample study. Facial spasm in different areas might have different incidence rate and progression rate. More possible factors related to progression rate might be missed. Therefore, more factors need to be included in the future, such as smoking, drinking, preoperative carbamazepine intake history, botulinum toxin injection history etc. Secondly, some patients with long medical histories might have experienced recall bias during the data collection process. A more detailed assessment scale of progression rate and a more reasonable rating of the severity of hemifacial spasm are needed in the future research.

## Conclusions

We have found that the rate of progression in hemifacial spasm was closely related to age of onset, facial nerve angle, degree of brainstem compression, and expression of APOE ε4. Specifically, patients with an onset age >45 years, facial nerve angle ≤ 96.5°, severer compression of the brainstem by offending vessels, and positive expression of APOE ε4 exhibited a faster progression rate. Based on the prediction of spasmic progression rate, clinicians and patients could choose an appropriate surgical timing for HFS.

## Data availability statement

The raw data supporting the conclusions of this article will be made available by the authors, without undue reservation.

## Ethics statement

The studies involving humans were approved by the Ethical Committees of The First Affiliated Hospital of Chongqing Medical University. The studies were conducted in accordance with the local legislation and institutional requirements. Written informed consent for participation was not required from the participants or the participants' legal guardians/next of kin in accordance with the national legislation and institutional requirements.

## Author contributions

FX: Conceptualization, Methodology, Writing—original draft, Formal analysis. PG: Data curation, Investigation, Writing—review & editing. HY: Investigation, Software, Writing—review & editing. LJ: Resources, Validation, Writing—review & editing. YX: Validation, Writing—review & editing. QS: Validation, Writing—review & editing. YZ: Conceptualization, Project administration, Supervision, Writing—review & editing.
